# On migration, geography, and epistemic communities

**DOI:** 10.1186/s40878-020-00193-2

**Published:** 2020-10-02

**Authors:** Russell King

**Affiliations:** Department of Geography, School of Global Studies, University of Sussex, Brighton, BN1 9SJ UK

**Keywords:** Migration studies, Interdisciplinarity, Geography, Epistemic communities

## Abstract

This commentary paper starts by questioning the assumption that migration means international migration, and goes on to affirm that migration studies has indeed come of age as a coherent if highly diverse research field. Several emerging epistemic communities are identified: migration and development; gender and migration; lifestyle migration; and youth and student migrations. Finally, I argue that the role of geography in the study of migration has been under-valued.

## Introduction

In this commentary on the important paper by Levy, Pisarevskaya, & Scholten (2020), I focus on four main reactions.

1. What are the core issues in the study of migration as a research field, and what are the boundaries of this field?
2. Has migration studies ‘come of age’? Where has it come from, and where is it going?
3. Are there some emerging ‘epistemic communities’ within migration studies which are not recognised in the analysis of Levy et al.?
4. Has the contribution of the discipline of geography been under-recognised?

As this piece is essentially a personal reflection on the nature and development of a research field, I shall partly rely, perhaps excessively, on some of my previous writings. These are built on several decades of research into migration in various parts of the world, but mainly in Europe, and on two meta-research roles which I have fulfilled. The first of these is the design and teaching of a postgraduate course, ‘Theories and Typologies of Migration’, which was the core course of the MA in Migration Studies at the University of Sussex between 1997 and 2011. The second was editing the *Journal of Ethnic and Migration Studies* from 2001 to 2013.

## Core issues in the study of migration

It is surprising how many of the key textbooks on the subject are reluctant to offer a concrete definition of migration (studies). Here is mine. *Migration* is the movement of people from one place to another, more distant place (i.e. not a ‘local’ move) and their residence there for a certain threshold of time (e.g. 6 months or 1 year). *Migration studies* is the broader study of this phenomenon: the description, analysis and theorization of the movement of people from one place, region or country to another – once again recognizing that there are thresholds of space and time built into this definition. Such moves should go beyond residential relocation within a village or town, and the moves should be longer than short-term visits, for instance for touristic or business purposes. The temporal threshold is commonly set at 1 year, but there are arguments for a shorter time-frame to recognize the relevance of seasonal and circular migration (King 2012c, p.7).

When teaching ‘Theories and Typologies of Migration’, I build my, and hopefully the students’, understanding of the field around five key questions enunciated 40 years ago by White and Woods (1980a, p. 1):

1. Why does migration occur? (This is the fundamental ‘theory’ question).
2. Who migrates? (And by the same token, who does not, and why not?).
3. What are the patterns of origins and destinations of migration flows and how have they evolved over time? (This is a basic question about the history, geography and statistics of migration).
4. What are the effects of migration on the places, societies and countries that migrants come from? (This question, above all, presages the recent debate on the so-called migration-development nexus).
5. What are the effects of migration on the places, societies and countries that migrants settle in? (This question’s most obvious links are to issues of ‘integration’).

Finally I add a sixth question:

6. What are the effects of migration on the migrants themselves? (This asks what is it like to be a migrant, or ‘migrancy’ as a state of being).

At first sight, these key questions, which in my view remain as valid today as they were in 1980, do not map easily onto the analysis carried out by Levy et al. on the evolving structure of the migration studies field. The relationship is clearest in the early years (1975–1984; see Levy et al., 2020, Fig. 7), whereby Demographers can be seen to be working on answers to questions 2 and 3, Economists on questions 1 and 4, and Assimilationists and Economic Sociologists on question 5. For later time periods, the relationships become less clear, as the dominant epistemic communities on the left side of the bubble diagrams – on Acculturation, Ethnic and Race Relations, Ethnic Entrepreneurship, and Migration and Health – increasingly reflect a reconfiguration of the field of migration studies as being about migration *and integration*, and only about *international* migration. These two changes imply on the one hand a narrowing of the field, ignoring internal migration, and on the other a broadening of the field by analysing not only the act of migration but also post-migration impacts after settlement in the destination country.

Both of these conceptual trends are flawed, in my view. It seems that nearly all of the analysis of Levy et al. is predicated on the definition of migration as *only* being about international migration. This, perhaps unwittingly, reflects the acronym of IMISCOE (International Migration, Integration and Social Cohesion in Europe), as well as the tendency of so many of the key textbooks on migration to be only about international migration, even though internal migration is globally much more widespread and numerically more important. Hence, Castles and Miller’s blockbuster *The Age of Migration* (first published, Castles and Miller 1993; latest edition, de Haas et al. 2020) is really ‘the age of international migration’, with little mention of internal migration. Likewise Brettell and Hollifield’s highly regarded *Migration Theory* (latest edition, Brettell and Hollifield 2015) is only about theories of international migration, with no reference to internal movements. And thirdly (although the examples could be multiplied) the textbook entitled *Key Concepts in Migration* (Bartram et al. 2014), which one might expect to have a broader remit, self-declaredly focuses almost exclusively on international migration. As I and my Sussex colleague Ron Skeldon have strongly argued (King and Skeldon 2010), this ‘gap’ between international and internal migration needs to be bridged, at both a theoretical and empirical level. Theoretically there is significant creative potential for integrating theories of internal and international migration; for instance regarding the causal factors for migration, the relationship between migration and development, or studies of cultural adaptation in the receiving-society context. Moreover, many migrants move *both* internally and internationally, and this simple fact needs to be empirically explored in more detail (for an excellent example, see Vullnetari 2012).

Secondly, the focus on the clusters of epistemic communities which examine the post-migration experience of international migrants, referencing keywords such as assimilation, acculturation, ethnic entrepreneurship, and race relations, seems to impose a straightjacket around how we think about migration. The presence of the word ‘integration’ in the IMISCOE acronym again probably plays a role here; but we should recognize the fundamental moral and philosophical doubts that exist concerning the use and instrumentalization of this concept, including its hegemonic and neo-colonial implications. These issues have been thoroughly and critically discussed in a recent debate in this journal led by Schinkel (2018, 2019), who labels ‘integration’, as a concept, ‘indefensible’. I return to this remark briefly in the Conclusion.

## Has migration studies come of age?

The answer to this question seems self-apparent, with evidence from multiple sources. The first is the sequence of colourful figures compiled by Levy et al. (see Figs. 7–10). What’s not to like about these beguiling multi-coloured bubble diagrams? From a picture of strung-out and fragmented clusters in 1975–1984 and 1985–1984, the pattern becomes a coherent, consolidated mass in 2005–2014, indicating that ‘a new age of migration studies has emerged’ (Levy et al., 2020).

What is also interesting is the array of key names around which the subcluster epistemic communities are articulated in terms of citation links and frequencies. Remarkable is the enduring centrality of Portes at the heart of the shifting sea of coloured bubbles, whilst the other subcluster leads – Berry for the Acculturationists, Borjas for the Economic Sociologists, Castles and Sassen for the Global Systems School – come as no surprise. What I do find initially surprising is the centrality of Bourdieu and Foucault in the Ethnic/Race Relations cluster, since they are not generally known as migration scholars. Rather, of course, they are cultural sociologists whose concepts and theories have been picked up by migration scholars who frame their work around notions such as ‘field’, ‘habitus’, ‘forms of capital’, ‘discourse’, ‘power’, ‘governmentality’ and so on.

The second part of the answer to this ‘coming of age’ question is provided by the qualitative material presented in Levy et al. (2020), and in another paper by the same research team (Pisarevskaya et al. 2019). This evidence comprises testimonies from the expert interviewees, the growth in the number of dedicated journals on migration, and the foundation of many research institutes and postgraduate degree programmes. Through all these lenses we can observe a rapid, at times accelerating, though not exponential (a statistical term that is often misused) increase in activity. Thus it seems to me beyond doubt that migration studies has come of age. Indeed, I wrote a paper with that very title 5 years ago in which I concluded: ‘no longer on the margins of the social sciences…migration has come of age as an academic field’ (King 2015, p. 2370).

A trickier question to answer is about the trajectory of migration studies: what are its origins and where is it now heading? Levy et al. (2020) advance the idea that migration studies emerged from the shadows of ethnic and racial studies in the 1970s–1990s. This is a perspective that I do not recognize: for me the origins lie much further back in classic writings by sociologists, geographers and economists, starting with Ravenstein (1885, 1889) and proceeding via important contributions by Thomas (1954, 1972), Sjaastad (1962), Lee (1966), Jackson (1969), Mabogunje (1970) and Zelinsky (1971) – to name but a very few of the notable pioneers. The future direction of travel hinges on the strength of migration studies as a vibrant interdisciplinary field of research, scholarship and teaching, its popularity amongst students and young researchers, its social and political importance, and generous funding schemes for collaborative research. Given the philosophical and methodological trends towards unity across the social sciences, the field of migration studies is routinely referred to as interdisciplinary, multidisciplinary, transdisciplinary, cross-disciplinary or postdisciplinary. This is not the place to tease out the differences (to the extent that they exist) between these labels; but rather to emphasize the strength, coherence and relevance of the rich field of migration studies.

## What are the emerging epistemic communities within migration studies?

Levy et al. (2020) define epistemic communities as groups of scholars who congregate around certain themes, concepts, disciplines or methods, creating ‘discursive spaces’ characterized by intensive cross-referencing. In their series of bubble diagrams based on bibliometric analysis and co-authorship, where each point represents a single well-cited author, these ‘communities of migration practice’ are drawn broadly as ‘schools of thought’, such as ‘Acculturationists’, ‘Global Systems School’, ‘Michigan/Wisconsin School’ etc., which are rather stable from one decade to the next. Underlying the evolution of epistemic communities mapped across the period between the 1970s and the 2010s is the enormous growth in the volume of publications, the steady growth in the proportion of co-authored papers, and the shift in the pattern of national origins of co-authored migration scholarship from one dominated by the USA to one where European, especially ‘English’, authors match the nodal power of the United States (Levy et al., Fig. 6). Of course, the countries mapped in Fig. 6 do not necessarily coincide with the nationality or country of provenance of the authors recorded, but merely the locations of the academic institutions where the authors are based. And behind this non-conformity lies a separate ‘story’ of the migration of scholars and researchers who have written about migration.

Undoubtedly there is more scope for fleshing out the interpretation of the series of co-citation maps (Levy et al. 2020, Figs. 7–10). For instance, what happened to the community of Refugee Studies scholars who were a clear cluster in 1975–1984, but then disappear as an epistemic community? We know that Refugee Studies remains a coherent and distinctive field of study with its own journals, research programmes and institutes. The most recent diagram, for 2005–2014, is timely to catch the emergence of the ‘Mobilities turn’ in the mid-2000s, with the foundation of the journal *Mobilities* in 2006, and the eponymous book by Urry (2007).

But which new epistemic communities are missing, and which are likely to grow further in the coming years? At least for Europe, how have recent key events, like the global economic crisis which struck in 2008, the so-called ‘refugee crisis’ of 2015–2016, or the coronavirus pandemic of 2020, affected evolving lines of migration research? Is the diversification of forms of mobility and the increasing relevance of ‘mixed migration’ flows and motives leading to a ‘refragmentation’ of the field of migration studies? One way to begin answering this array of questions is to look at IMISCOE as the largest and most vibrant network of European and global migration scholars and at the way in which its business is organized into research clusters and initiatives, and how these themes have changed over the 15 years of the Network’s existence.

The original configuration of IMISCOE’s nine research clusters contained several which do not fit neatly onto the bubble maps and which continue to be the focus of important research globally. One of these is *Migration and Development.* Historically, this relationship has been analysed mainly through a ‘first world’ optic (Thomas 1954; Zelinsky 1971), but with the landmark notion of the ‘migration-development nexus’ (van Hear and Sørensen 2003), this relationship now mainly focuses on the multiple ways that migration can aid development in the migrant-origin countries, for instance through the inflow of remittances. However, this is only one interpretation of the migration-development nexus: an alternative view sees it in a much more negative light, due to brain drain and dependency relationships (de Haas 2010). This leads me to make another, more general point about the ‘migration studies mapping’ achieved by Levy et al., which is the omission of perspectives from the global South (Castles and Delgado Wise 2008). When migration origins and destinations are combined, the global South outscores the global North, as well as being the place where the largest-scale internal migrations are currently taking place.

A second IMISCOE thematic cluster ‘missed out’ on the bubble maps was on *Gender, Age and Generations.* At first sight, this could be seen to approximate the Demography cluster mapped on Figs. 7–10, but Levy et al. interpret the demographics of migration largely through the lens of the quantification of population change. Instead, this IMISCOE cluster has spawned, and reflected, other important lines of research which, arguably, constitute concretely existing or embryonic epistemic communities. The most important of these is surely *gender and migration,* which has evolved a vibrant and well-connected literature, as well as gender being increasingly mainstreamed into most other branches of migration research (for a variety of insights see Gabaccia and Donato 2015; Pessar and Mahler 2003; Willis and Yeoh 2000).

Two other types of migration also became paradigmatic around the turn of the millennium, and are well-represented in recent IMISCOE research cluster activities. Both reflect migration drivers that are less overtly economic (as with labour migration) or political (the case of refugees) and more to do with life-stage and lifestyle. *International student migration* has received a lot of attention, mainly from younger scholars who, in most cases, have had their own experiences of international academic mobility (see for instance Bilecen 2014; Van Mol 2014; and for overviews of the field King and Findlay 2012; King and Raghuram 2013). International students are not conventionally seen as ‘migrants’ (even if technically they usually are), and are part of a wider phenomenon of *youth mobilities* where education, adventure and lifestyle are generally predominant over strictly economic migration motives (King 2018).

Secondly, another vibrant community of scholars unites prolifically about the overlapping phenomena of *international retirement migration* and *lifestyle migration,* where a better ‘quality of life’ is sought in a pleasant scenic and climatic environment. Within Europe, these migrations, which can be either seasonal or permanent, are from the cold-winter climates of the north to warmer and sunnier coasts and rural areas of the south, with extensions to Turkey and Morocco, as well as further beyond to Thailand and the Caribbean. Parallel North-South lifestyle and retirement migrations are observable in the Americas: from the USA and Canada to Mexico, the Caribbean, Ecuador and elsewhere. The pioneering European studies are by Benson (2011) and O’Reilly (2000); see King (2012b) for an overview.

Finally, if an epistemic community can be based around a methodological ‘turn’, then the rapid growth in in-depth qualitative studies of migration constitutes a powerful element which strengthens both the volume and insight of papers in recent decades. This development is coincident with the ‘cultural turn’ in migration research, as Levy et al. (2020) note. The quantity of citations to Bourdieu, Foucault and Stuart Hall are indicative of this development, tying the cultural approach to the Ethnic/Race Relations theme (Figs. 9 and 10), but in reality the impact of qualitative methods is much wider across the field of migration studies (Zapata Barrero and Yalaz 2018), including in more recent years a ‘sexual turn’ (Mai and King 2009) and an ‘emotional turn’ (Boccagni and Baldassar 2015; Svašek 2010).

## Has geography’s contribution to migration studies been under-valued?

The fourth and final question involves me riding a personal hobby-horse. As a phenomenon that unfolds across space (and time) migration is quintessentially geographical. Yet, I believe that geographers’ contributions to the study of migration have been consistently under-appreciated (King 2012a), and this impression is not allayed by reading the paper by Levy et al. (2020). Why do I feel this way? In the past it might have been due to a collective sense of inferiority amongst geographers. Caught between the physical sciences on the one hand and the humanities and social sciences on the other, geography seemed to be a subject that was somehow ‘lost’ on the academic playing field, where it was seen as a descriptive discipline of facts, figures and regional monographs. Certainly it lacked a critical edge. David Harvey and Doreen Massey did much to restore ‘respect’ for geography, whilst the climate crisis and other pressing environmental issues created the need to reunite physical and human geography, and the physical and social sciences more broadly, to respond to these global challenges. Nowadays geographers studying migration can feel inspired by the words of Favell (2008, p. 262) when he describes ‘geography… [as] arguably the most exciting discipline in the social sciences’.

Yet geography gets rather short shrift by Levy et al. In their discussion of the historical development of migration studies, sociology is confirmed as having prime importance, followed by economics and demography, as the three foundation disciplines, No mention is made of geography, nor of the fact that Ravenstein, ‘father-figure’ of the study of migration, was a geographer. In the citation cluster diagrams, economics, sociology, demography and psychology are referenced, but not geography or anthropology, indicating that the contributions of these two disciplines are marginal. And in regard to the series of quinquennial coloured ‘discipline’ diagrams in Fig. 11, Levy et al. argue that (social) geography only became visible as a constitutive discipline of migration studies since 2010, mainly functioning as a lynchpin of interdisciplinary research.

One of the probable reasons why geography makes so little visible impact on the diagrams and analysis in the Levy et al. paper is that geographers have not worked in harmony to produce a ‘school’, but have instead made their contributions across many sub-fields of migration studies. My guess is that the significant quantitative work done by population geographers is hidden in the Demography cluster, whilst economic, social and cultural geographers have spread their research around most of the intellectual territory mapped by the bubble diagrams, contributing to ethnic and racial studies, acculturation, and global systems, as well as to migration and development, issues of migrant identity, transnationalism, diaspora, the mobilities paradigm, and many more. This eclecticism can be argued to be both a strength and a weakness; for me it is a strength! One simple and near-to-home example of geographers’ impact on the institutionalization and management of migration studies comes from the original structuring of IMISCOE into nine research clusters, four of which were led by geographers.

Geographers have also authored a string of state-of-the-art textbooks on the theory and structure of migration, indicating their skills at synthesis, comparison, and combining local, regional and global views of the phenomenon. Amongst the most significant of these are, in chronological order, White and Woods’ (1980b) *The Geographical Impact of Migration,* Skeldon’s (1997) *Migration and Development,* Boyle et al.’s (1998) *Exploring Contemporary Migration,* Samers’ (2010) *Migration* (second edition, Samers and Collyer 2017), Mavroudi and Nagel’s (2016) *Global Migration,* and the very recent *Handbook on Critical Geographies of Migration* (Mitchell et al. 2019). For my money, the Boyle et al. text still represents the most comprehensive overview of the field of migration. It is especially strong on internal migration, and covers both the more technical and economic, as well as the social and cultural, aspects of migration theory and literature. A pity that a newer edition has not been produced. Meanwhile, the Mitchell et al. *Handbook* represents a brilliant and comprehensive overview of the advanced state of critical geographical scholarship on a wide range of migration themes, including embodied and gendered geographies of migration, geographies of borders and migration control, diaspora and transnationalism, and refugees and humanitarianism. Above all, geographers’ sensitivity both to theorizing migration ‘from above’ and, more especially, to an emic interpretation of the migrant condition helps to answer the sixth key question posed much earlier in this commentary, about the effects of migration on migrants themselves.

## Conclusion

The paper by Levy et al. is an original and fascinating exploration of the development of the interdisciplinary field of migration studies, based on multiple methods and sources, with a huge literature-crunching exercise at its core. Migration scholars will learn much from this paper, but it also raises as many questions as it answers. For instance, why does Transnationalism not emerge as a prominent epistemic community, when this has been arguably the dominant paradigm in the study of international migration for the past 25 years? Likewise the significant contributions of political science and the migration policy community are screened out of the analysis, whilst geography and anthropology are marginalised in favour of sociology, economics and demography. I understand that ‘this is what the analysis shows’, but intuitively I feel that the results are somewhat out of focus with the reality of the research field of migration studies. It is also out of sync with the reality of life for most migrants, which is more about migration as a reaction to, and embodiment of, poverty and inequality.

In answer to one of the key questions posed by Levy et al., whether migration studies has come of age, I concur, but with the proviso that migration has diversified. Perhaps, instead, we have a new age of diversified mobilities. In answer to another key question – whether the growth of migration studies leads to institutionalization and consolidation of the research field or to fragmentation and the proliferation of new sub-fields – I respond that both are going on simultaneously. Comparing Levy et al.’s Fig. 7, with Fig. 1, consolidation appears dominant, but at the expense of excluding new epistemic communities which have yet to reach the threshold to register on the diagrams.

My next concluding point is to speculate on how interesting it would be to apply the cluster mapping technique to other subjects, either established disciplines such as sociology, geography or economics, or to interdisciplinary fields, where Development Studies comes to mind as an obvious candidate.

Finally, it should be acknowledged that there is a fundamental critique of migration (and integration) studies out there. Amongst many migration scholars, there is an undeniable tendency to categorize, essentialize and even fetishize migrants, thinking that their migration is as vitally important for ‘them’ as it is for ‘us’ as scholars of ‘their’ migration. We need to remove the fiction that, for the people we label and objectify as migrants, migration is the most important event in their lives – often it is not (Rogaly 2015). Likewise, Dahinden (2016) pleads for the ‘de-migranticization’ of migration and integration research and its nation-state and ethnicity-centred epistemologies. Instead we should ‘re-migranticize’ social-science research in general, bringing the everyday realities of migrancy, race and ethnicity into the centre of social theory. Even more trenchant in his critique is Schinkel (2018, 2019); his rapier-like words slashing into the academic edifice of migration and integration studies. Migration studies, in his view, is an ‘imposition’ which naturalizes and objectifies migrants, and integration is seen as an indefensible neo-colonial exercise geared to the political and socio-cultural hegemony of the nation-state and – here is the bit that really hurts – to academics’ career-building. What this means for the CrossMigration project, I leave to its leading members to respond.
